# American Medical Association

**Published:** 1880-07

**Authors:** 


### Editorial.

---

### AMERICAN MEDICAL ASSOCIATION.

In another part of this number of the JOURNAL AND EXAMINER will be found a very full and excellent report of the proceedings of the recent meeting of the American Medical Association, in the city of New York. It appears to have been a very full, harmonious and interesting meeting. The only objections we have seen or heard concerning it, arose from a mistake on the part of the Committee of Registration, in providing inadequate accommodations for the prompt registration of members, and from a serious neglect to pay proper attention to the by-laws and regulations concerning the arrangement of papers and the preparation of a proper programme for the whole meeting, and the proper examination and disposal of the papers in the several sections. It is one of the curious things in human experience to note the frequency with which men selected expressly to perform certain official duties which are plainly set forth in constitutions and by-laws, fully accessible to them, go about the work assigned to them without taking the slightest pains to read even such by-laws as are particularly designed to guide their own action. For instance, the by-laws of the Association were so changed two years since as to require all persons proposing to present reports or read papers either in the general meetings or in the sections, to send such papers, or abstracts of the same, to the chairman of the Committee of Arrangements, at least one month before the meeting of the association, expressly for the purpose of enabling such committee to examine and arrange the same and make up a full programme for the annual meeting. Yet, entirely overlooking this plain provision, the committee in New York took pains to give special notice through the medical journals that all reports and papers must be sent to the officers of the several sections, scattered throughout the Union, at the very time that they should have been sent to the committee in New York.

Of course they had no opportunity to make up and print a full progrramme, to be given each member when he registered his name, as the by-laws intend shall be done. We hope the Committee of Arrangements for the next meeeting, in Richmond, will at least read the by-laws on this subject, as published in the *Transactions* for 1879. In regard to the proper disposal of papers in the several sctions, the by-laws and ordinances, published in the *Transactions* every year for the last dozen years, give specific and plain directions. First, they positively prohibit the reference of any paper to the Publication Committee until it has been actually read and considered in the section, or by a sub-committee appointed by the section for that purpose.

Second, they require all papers read in the sections to be placed in one of three classes, namely, such as contain the results of *original* experiments and investigations of value ; such as contain so complete a résumé of what is known on a subject of importance that it leads to the establishment of practical conclusions of real value ; and such as contain more or less matter of importance, of an isolated or fragmentary character. Only papers belonging to the two first classes are allowed to be referred to the Committee of Publication, while those of the third class are to be referred back to their authors with permission to publish them in any medical periodical they may choose.

The rules further provide that any paper, either from its length or from want of time, which cannot be read and properly considered by the section, must be referred to a sub-committee of the section, with thirty days for examination and a report to the Committee of Publication.

It is thus seen, that if the officers of sections who have a whole year in which to learn the regulations of the Association, before they are called on to act, would give even a very moderate attention to the rules printed in every volume of *Transactions*, there could be no such thing as reading papers by their titles and referring them blindly for publication in the *Transactions*, as was done at the recent meeting, and has been often done before.

We have alluded to these existing regulations because some of our cotemporaries are calling for *better* regulations to cure the evils complained of; whereas, the fault is not in the *regulations*, but in the neglect to enforce them. We suggest that the best way to remedy the evils complained of, and to render the working of the sections more efficient and valuable every way, would be to make the secretary of each section a permanent officer instead of an annual one. Let a good, firm and enterprising member hold the office of secretary from year to year, and having once learned the regulations and modes of work, he would easily keep all proper rules before the meetings and secure their observance, much to the pleasure and profit of all parties. D.

